# Retinal Biomarkers in Diabetic Retinopathy: From Early Detection to Personalized Treatment

**DOI:** 10.3390/jcm14041343

**Published:** 2025-02-18

**Authors:** Georgios Chondrozoumakis, Eleftherios Chatzimichail, Oussama Habra, Efstathios Vounotrypidis, Nikolaos Papanas, Zisis Gatzioufas, Georgios D. Panos

**Affiliations:** 1 Department of Ophthalmology, University Hospital of Heraklion, 71500 Heraklion, Greece; 2 Department of Ophthalmology, University Hospital of Basel, 4031 Basel, Switzerland; 3 Department of Ophthalmology, Ulm University, Prittwitzstraße 43, 89075 Ulm, Germany; 4 Diabetes Centre, Second Department of Internal Medicine, Democritus University of Thrace, 68100 Alexandroupolis, Greece; 5 First Department of Ophthalmology, AHEPA University Hospital, School of Medicine, Aristotle University of Thessaloniki, 54124 Thessaloniki, Greece; 6 Division of Ophthalmology & Visual Sciences, School of Medicine, University of Nottingham, Nottingham NG7 2UH, UK

**Keywords:** diabetic retinopathy, retinal biomarkers, biomarkers, diabetes, retinal imaging, ophthalmology, diabetes complications

## Abstract

Diabetic retinopathy (DR) is a leading cause of vision loss globally, with early detection and intervention critical to preventing severe outcomes. This narrative review examines the role of retinal biomarkers—molecular and imaging—in improving early diagnosis, tracking disease progression, and advancing personalized treatment for DR. Key biomarkers, such as inflammatory and metabolic markers, imaging findings from optical coherence tomography and fluorescence angiography and genetic markers, provide insights into disease mechanisms, help predict progression, and monitor responses to treatments, like anti-VEGF and corticosteroids. While challenges in standardization and clinical integration remain, these biomarkers hold promise for a precision medicine approach that could transform DR management through early, individualized care.

## 1. Introduction

Diabetic retinopathy (DR) is the most common microvascular complication of diabetes mellitus (DM) and the leading cause of visual impairment and blindness in working-age populations globally [1,2]. The disease is characterized by an initial, non-proliferative stage (NPDR) that manifests with increased vascular permeability and ischemia. NPDR can progress into proliferative DR (PDR), when excessive ischemia leads to neovascularization and subsequent life-threatening complications, such as vitreous hemorrhage and tractional retinal detachment [3]. The involvement of the macula can occur at any stage of NPDR or PDR, mainly as diabetic macular edema (DME) [4].

DR prevalence in the diabetic population is approximately one-third of all cases, accounting for 126 million patients globally [1,5]. DME is the leading cause of moderate visual loss, especially among diabetes type 2 patients, while PDR is the most common type of vision-threatening lesion in type 1 diabetes [6]. Most of the people with type 1 diabetes exhibit some form of DR in their lifetime, while the respective risk among type 2 diabetes patients is 50–60% [7]. However, in people with type 2 diabetes, diabetic retinopathy can be present directly at diabetes diagnosis, possibly due to years of undiagnosed diabetes [8].

The determination of the various stages depends on microvascular-related clinical signs, such as microaneurysms, hemorrhages, and exudates [3]. Complementing clinical examination, optical coherence tomography (OCT) allows clinicians to detect macular edema in patients before retinal thickening is visible on slit lamp microscopy, while fluorescein angiography (FA) represents the mainstay to detect neovessels [4,9]. According to recent studies, DR is not only a diabetic microvascular complication but also a neurodegenerative disease [10]. However, the current staging systems of DR are based on clinically detected microvascular changes that are unable to detect neurodegenerative lesions that might often be already present in newly diagnosed diabetes [11].

Although there is no doubt regarding the relationship between established risk factors, such as glycemic control and the progression of DR, recent data indicate that HbA1c values explained only up to 11% of the risk of DR among patients and that the remaining variation in risk is attributed to other, yet unidentified, elements [12]. A similar variation has been observed regarding the individual response to treatment, as clinical trials suggest that anti-VEGF agents for DR do not display equivalent efficacy in all patients [13]. These observations point to the need for a deeper understanding of the pathogenesis of diabetic retinopathy, as well as the future therapeutic targets.

Given the increasing incidence and prevalence of diabetes and our limited capacity to screen and treat diabetic retinopathy, there is a need to reliably identify and triage people with diabetes. In this context, the identification of ocular and systemic biomarkers is crucial to facilitate the early diagnosis and to guide the risk stratification of diabetic patients, ensuring timely intervention before substantial structural damage and sight-threatening complications. Moreover, biomarkers may also guide treatment choice and improve the monitoring of the response to treatment, improving our ability to assess each patient individually. This includes the identification of subgroups of patients with diabetes and retinopathy, according to their different responses to treatment, a common practice in other areas, such as oncology, but not yet in diabetes. The primary aim of this paper is to review published literature regarding the ongoing research for novel soluble and diagnostic imaging biomarkers for diabetic retinopathy.

## 2. Definition of Biomarkers

In the past, the term biomarker was defined as a characteristic that is objectively measured and evaluated as an indicator of normal biological processes, pathogenic processes, or pharmacologic responses to a therapeutic intervention. In contrast to clinical findings, biomarkers refer to a broad subcategory of any substance, structure, or process that can be measured accurately and reproducibly [14]. Although the term commonly points to a molecular target, in fact, every molecule, physiologic and clinical measurement, and histologic or imaging finding can serve as a biomarker [15]. However, the terms biomarker and risk factor are often used interchangeably. A risk factor can be defined as any attribute, characteristic, or exposure of an individual that increases the likelihood of developing a disease or injury [16].

Consequently, a biomarker is a quantitative characteristic, which can not only be utilized as a risk factor but has a much broader spectrum of implementations, such as early disease detection, risk for progression assessment, individualized treatment choice, and the monitoring of response to treatment [15]. Molecular biomarkers in particular may also facilitate a better understanding of the pathogenesis of diabetic retinopathy and contribute to the development of novel treatments and new clinical strategies to prevent vision loss in people with diabetes [17]. In most cases of molecular biomarkers, there is evidence that the molecule under investigation is involved in the process of a key pathway stage of the disease. However, adopting this assumption in all cases imperils mistaking correlation for causation [14].

Nowadays, with the wide application of omics techniques, multiple biomarkers emerge as predictive and therapeutic targets for diabetic complications, and increasing potential agents are in clinical trials or undergoing preclinical investigations [18]. New analytical techniques, in particular those related to molecular markers, are accelerating progress in diabetic retinopathy research [17]. Furthermore, artificial intelligence (AI) is also developed and has been implicated in precision medicine, which along with improved imaging techniques could facilitate the improvement of diagnosis and outcomes of microvascular complications [19].

## 3. Methods

A comprehensive literature review was performed regarding the biomarkers of diabetic retinopathy using the databases PubMed and Scopus. Given the vast field of the ongoing research for DR biomarkers, each category of either imaging, genetic, or ocular and systemic soluble biomarkers was studied separately. The keywords used for this search were ocular biomarkers diabetic retinopathy; serum biomarkers diabetic retinopathy; imaging biomarkers diabetic retinopathy; and genetic biomarkers diabetic retinopathy.

We included primary preclinical and clinical studies with both prospective or retrospective design and reviews; whereas, case reports, observations, expert opinions, and letters to the editor were excluded. No restrictions were placed upon our search in terms of the year of publication; however, the most recent studies for each category of biomarkers were prioritized, as the main focus of this study is to provide an update about the relevant ongoing research. During the whole process, all the eligible articles were meticulously studied, and data were collected by two authors, GC and LC, working independently.

Further categorization was implemented according to the sample tissue or the physiologic action for soluble biomarkers, as well as the specific imaging technique for imaging biomarkers. A secondary search took place for each specific biomarker, following the primary assessment of the literature. Finally, all of the biomarkers studied were summarized in terms of their intended clinical use in screening, risk assessment, therapy response monitoring, and individualized therapy selection.

## 4. Molecular/Soluble Biomarkers

Primary studies among the literature have examined the presence of potential biomarkers in different biological fluids, such as vitreous humor, aqueous humor, and blood. Glycosylated hemoglobin (HbA1C) is currently the only soluble serum biomarker with clinical use [12,20], while most of the potential soluble biomarkers are in the preclinical stage of evaluation.

### 4.1. Sample Tissue

Vitreous humor (VH), aqueous humor (AH), and tears have been used to detect novel biomarkers; although, there are no universally validated local biomarkers for clinical application, currently. Circulating biomarkers in the serum display many advantages, such as a greater sample volume and availability with well-established routine collection procedures and analytical techniques with high repeatability [21]. However, as retina is a small tissue relative to the total body mass, serum biomarkers for DR related to local pathophysiology should display high specificity in order to be clinically relevant [21,22].

#### 4.1.1. Vitreous Humor

Vitreous humor is the most appropriate tissue sample for the detection of biomarkers, reflecting the pathological processes taking place in DR, due to its tight proximity to the retina [23]. Vitreous collection—often referred to as liquid biopsy—is an invasive procedure requiring pars plana vitrectomy, and is, thus, not widely available for research in asymptomatic patients, when a therapeutic intervention is not indicated [23]. However, vitreous tap office-based aspiration has also been performed as a possible safe alternative [24]. Additionally, the dilution of the sample taking place intraoperatively should also be taken into consideration [25].

#### 4.1.2. Aqueous Humor

On the contrary, aqueous humor collection is a less invasive way to obtain clinically useful biomarkers, which can even be performed on an outpatient basis during slit lamp examination [26]. The low volume of ocular samples from vitreous and aqueous humor, as well as the aforementioned limitations, indicate the importance of the standardization of collection procedures, sample storage, and analysis in order to be applicable in DR decision making in the future [21].

#### 4.1.3. Tears

Tear collection, on the other hand, could be a completely non-invasive alternative source of biomarkers, in comparison to the aqueous and vitreous humor methods. It has been proposed as a promising body fluid for monitoring not only ocular diseases but systemic conditions also [27]. Conventional techniques, such as tear strips, cellulose sponges and capillary tubes, are time consuming and can induce discomfort, constraining every day clinical application [27,28]. Moreover, when conjunctival conduct is required for sample collection, the irritating stimuli could cause reflex tear overproduction diluting the baseline concentrations [27]. Therefore, recent advances in contact lens biosensor technology may allow for the real-time monitoring of tear biomarkers in the future [27,28]. Although tear biomarkers are mainly intended for anterior segment ocular diseases, such as dry eye disease and glaucoma [15], many studies have also examined the presence of useful biomarkers for diabetic retinopathy.

### 4.2. Analytical Techniques

Many types of biomolecules, like DNA, RNA, polypeptides, lipids, and metabolites, can serve as a biomarker. Various analytical techniques have been traditionally used for each type of molecule, like immunoassays for proteins [29]. The development of new high-throughput technologies, such as mass spectroscopy and next generation DNA sequencing, along with leaps in the improvement of big data analysis, has led to the emergence of the term multi-omics [18,29]. Omics is a discipline of science for analyzing interactions between molecules, following the direction of information all the way through from DNA to proteins. Genomics, epigenomics, transcriptomics, proteomics, and metabolomics constitute some of the respective subcategories of the omics techniques [18]. The last two are of greater importance for novel biomarker development [29].

Multi-omics techniques have revolutionized our ability to detect novel biomarkers and our understanding of their role in the disease mechanism, even within small volume samples [18]. Commonly, the alterations of a molecule in a disease reflect reactive rather than causative interactions, creating a vague impression about the underlying pathophysiology. The integration of various omics data provides a multilayered picture of the disease, which could improve our insight into the cascade of the underlying pathophysiology [18]. Moreover, recent advances in analytical techniques used along with mass spectrometry in proteomic analysis have enabled us to detect numerous post-transcriptional changes, improving our functional interpretation of these data [30]. Furthermore, bioinformatic software, such as STRING 12.0, allows for the mapping of the interactions between proteins, making the application of findings more useful [31].

However, many of these molecules are found in low concentrations, which can be masked by highly abundant molecules, like albumin and hemoglobin [32]. Column chromatography can be utilized in order to deplete molecules of higher concentration, facilitating the study of possible biomarkers, which otherwise could not be easily detected [32]. Given the variability in the concentration of specific molecular biomarkers among individuals, as well as the complexity of molecular interactions in multifactorial diseases, the use of a panel of biomarkers instead of single tests has gained increased popularity. The combination of multiple biomarkers in a single test has been shown to increase the overall accuracy and predictive value, in comparison with the use of a sole one [29].

Moreover, considering the multifactorial and complex nature of disease pathogenesis, many types of molecules, such as inflammatory, angiogenic, oxidative stress, metabolic, and neurodegenerative factors, could be appropriate candidates as biomarkers [3,4,10]. More importantly, many of the molecules are identified in both serum and ocular specimens used as samples, further highlighting the systemic and local involvement of various factors in the disease entity [32]. As such, these factors are presented separately according to their physiological role.

### 4.3. Types of Biomarkers

#### 4.3.1. Inflammatory Biomarkers

Inflammation possesses a pivotal role in diabetic retinopathy; thus, inflammatory mediators have been extensively studied in serum and ocular fluids. Wu published a series of studies assessing several inflammatory cytokines in the aqueous, vitreous, and serum of healthy subjects and patients with NPDR and DR [22,33,34]. Multiplex immunoassays were utilized in all three experiments to test multiple molecules at once. Multiple cytokines from the interleukin (IL) family, interferon-γ (INF-γ), tumor necrosis factor-α (TNF-α), and chemokines, like monocyte chemoattractant protein-1 (MCP-1), were found to be significantly higher in DR patients compared to the non-DR control group [22,33,34]. Additionally, the levels of many of them have been shown to differ between NPDR and PDR patients, pointing to their future use not only for early diagnosis but also for staging and disease progression monitoring [22,33,34].

However, the plasma levels of these cytokines were not significantly altered in comparison to their local concentrations, which is to be expected, considering the low systemic concentration of these messenger molecules even in generalized inflammatory states [22,35]. Thus, cytokines may not be a promising serum biomarker. C-reactive protein (CRP) could alternatively be used in the future as a biomarker for disease severity, indicated by the results of a systematic review by Song et al. [36]. Tear multiplex analysis by Amorim et al. has also revealed alterations in many cytokines [37].

Proteomic analysis, especially using mass spectrometry, has revealed numerous inflammatory mediators, like chemotactic factors, complement complex, adhesive molecules, coagulation, and growth factors, displaying altered levels in patients with DR [32]. The statistical analysis of a postmortem vitreous proteomic analysis of DM revealed various other candidate targets for future in vivo studies, such as inositol 1,4,5-trisphosphate receptor type 2 (ITPR2), calcium homeostasis endoplasmic reticulum protein (CHERP), and coronin-1A (CORO1A) [38]. However, the careful interpretation of the results is required, as proteomic analysis has many limitations, such as the failure to identify proteins in low concentrations in the presence of highly abundant ones [39].

Given the vast number of proteomic studies, most of which analyzed vitreous samples in patients with diabetic retinopathy, two systematic reviews by McAueley et al. and Manson et al. can demonstrate the most frequently identified molecules to be overexpressed in DR patients [40,41]. Other inflammation modulatory molecules, like long pentraxin 3 (PTX3) acting on complement factors, have also been proposed as possible biomarkers and therapeutic targets [42].

#### 4.3.2. Angiogenic Biomarkers

Several studies have previously reported a shift towards elevated pro-angiogenic mediators in DR, using traditional detection methods, like immunoassays and Western blot [43,44,45]. Placenta growth factor (PlGF), matrix metalloproteinase-2 (MMP-2) and matrix metalloproteinase-9 (MMP-9), angiopoietin-1 (Ang-1) and angiopoietin-2 (Ang-2), vascular endothelial growth factor (VEGF), erythropoietin (EPO), transforming growth factor-b1 (TGFb1), and thrombospondin-1 (TSP1) demonstrated higher levels in vitreous samples of DR patients, while the levels of EPO and VEGF were also higher in aqueous humor [43]. Interestingly, VEGF in serum was lower in diabetic patients [46]. Genomics analysis further supported the robust role of angiogenesis as a hallmark of DR. Angiogenesis-related genes, such as PIK3CB, ALDH3A1, ITGA7, FGF23, THBS1, COL1A1, MAPK13, and AIF1, have been associated with the early development of DR [47].

#### 4.3.3. Metabolic Biomarkers

One main disadvantage of proteomic studies is the absence of the direct correlation of the findings with phenotypes. DR is a primary metabolic disorder, and as such, metabolomics could be a more promising method with more precise clinical interpretation [20]. The primary measurement methods utilized in metabolomics are mass spectrometry (MS) and nuclear magnetic resonance (NMR). Different approaches are divided into untargeted metabolomics and targeted metabolomics [20]. The first approach aims to identify as many novel biomarkers as possible, while the latter aims to quantify and validate the role of each biomarker [20].

A systematic review by Hou et al. revealed changes in the levels of amino acids and various metabolites among ocular matrices, as well as in plasma of patients with DR [48]. Many further metabolites have been proposed in the literature through metabolomic analyses (Table 1) [49]. Non-enzymatic glycation is considered the main primary mechanism of damage in diabetes mellitus, leading to the accumulation of advanced glycation end products (AGEs), causing direct and indirect damage to tissues [50]. Therefore, levels of AGEs in plasma have been proposed as systemic biomarkers by many studies [50]. Studies into tears have also identified possible biomarkers for either early diagnosis or risk for PDR assessment [51,52].

#### 4.3.4. Oxidative Stress Biomarkers

Oxidative stress has long been speculated as a major component of the microvascular and neurodegenerative component of damage, mainly through membrane lipid peroxidation [61,62]. The impairment of basic metabolic pathways, such as polyol, advanced end glycation products (AGE), hexosamine, protein kinase C (PKC), and the tissue renin–angiotensin system (RAS), leads to oxidative burden [63]. Many studies have correlated single or a panel of oxidative biomarkers with the risk of developing DR or PDR using ocular matrices, such as tears, or by evaluating their plasma concentrations [64].

The role of malondialdehyde (MAD), a byproduct of lipid peroxidation [55,56], the total antioxidant capacity (TAC) [56], direct lipid hydroperoxidation (LPO) [55], total superoxide dismutase (SOD) [55], glutathione (GSH) [55], and nitric oxide (NO) [65,66], has been previously studied, utilizing various analytical methods. A proteomic analysis of tears has also identified many peptides with oxidative related functions in patients with DR (calmodulin-like protein 5 (CALML5), glutamine synthetase (GLUL), protein SET: protein SETSIP (SET/SETSIP), DNA dC->dU-editing enzyme APOBEC-3A (APOBEC3A), cathepsin L1 (CTSL), glutaredoxin-1 (GLRX), nicotinamide phosphoribosyltransferase (NAMPT), alpha/beta hydrolase domain-containing protein 14B (ABHD14B), protein disulfide-isomerase A3 (PDIA3), and calmodulin-like protein 3 (CALML3) [37].

#### 4.3.5. Neurodegenerative Biomarkers

As discussed earlier, DR is not only considered a microvascular complication of DM but a degeneration of the neurovascular unit. Glial cell dysfunction is believed to be involved in the neurodegeneration process, so the potential biomarkers indicative of Müller cell disorganization have been studied. The vitreous levels of neurotrophins have been found to be higher in DR eyes, supposedly as a responsive rescue attempt [53]. Additionally, aqueous analysis has demonstrated increased levels of glial fibrillary acidic protein (GFAP), aquaporin 1 (AQP1), and aquaporin 4 (AQP4), which is believed to be due to overproduction by glial cells [54].

#### 4.3.6. nc-RNA (Non-Coding RNA)

Non-protein-coding RNA molecules are involved in gene expression modulation [67]. miRNAs are short ncRNAs regulating gene expression at the transcriptional and post-transcriptional level. These molecules are characterized by long half-lives, making them ideal as biomarkers in ocular fluids [58]. Long ncRNAs (Lnc-RNAs) were larger recently discovered, with nc-RNA influencing gene expression through epigenetic interactions, transcription, and translation regulation [68]. Both categories of molecules have recently gained a lot of popularity as novel biomarkers [67].

The analysis of transcriptomic databases have allowed for the identification of nc-RNA molecules, as well as their respective genes [60]. Liu et al., through complex bioinformatics of pre-existing databases analysis, have identified OSER1, HIPK2, and DDRGK1 genes and their products as possible novel biomarkers in DR [60]. They further detected the altered expression of these molecules using RT-qPCR in the blood samples of DR patients. In a similar way, Grieco et al. profiled the AH and plasma of DR patients to detect miR-200b-3p, let-7c-5p, miR-365-3p, and miR-199a-3p molecules to be involved in DR pathogenesis [58]. Tear profiling has also demonstrated many miRNA molecules to be overexpressed with a potential use as biomarkers, while miR-218-5p levels, in particular, were correlated with disease progression [59]. Furthermore, considering mi-RNAs take part in the inflammatory and oxidative processes of DR development, it has been proposed as a novel therapeutic target, as well [69].

Moreover, lnc-RNA, such as Lnc-RNAs MEG3 and MALAT1, can be associated in DR and constitute future biomarker and therapeutic targets [57,68]. They can also facilitate the deeper understanding of the complex pathophysiology of DR; however, further research is required to clarify their role in DR [57]. In other fields of medicine, such as lung cancer, asthma, and myocardial infarction, much progress has been noted, offering a promising perspective for ocular diseases as well [68].

#### 4.3.7. Extracellular Vesicles

Extracellular vesicles (EVs) are secreted by almost any type of cell in the human body and contain proteins and genetic material, while playing a pivotal role in cell-to-cell communication, immune and inflammatory response regulation, and neovascularization [70]. EVs, which are believed to be involved in the development of many cancers and cardiovascular and neurodegenerative diseases, have recently drawn attention as possible biomarkers for ocular diseases [70]. EVs are speculated to alter the function of pericytes, taking part in the pathogenesis of DR [71].

EVs can be isolated from samples with centrifugation, and their physical properties are analyzed with ultra-sensitive flow cytometry called nanoparticle tracking analysis (NAT) [72]. Changes in the concentration, size, and proteomic profile of EVs have been detected in cadaveric retina samples [73]. Additionally, the miRNA content of EVs from serum samples analyzed with qRT-PCR differs between DM patients and healthy subjects, while serum EVs from DR patients have been able to induce DR-related vascular changes in in vitro experiments [72]. All these findings point to the possible future application of EVs and their content as DR biomarkers for early diagnosis, disease progression assessment, and possibly, as therapeutic implementations [74].

## 5. Imaging Biomarkers

Multiple imaging modalities have been extensively used in clinical practice in the evaluation of DR as complementary tools to indirect ophthalmoscopy [75]. The Early Treatment Diabetic Retinopathy Study (ETDRS), using stereoscopic fundus photos, set the gold standard for over half a century to guide the diagnosis, staging, and decision making of DR [76]. Diabetic macular edema was traditionally evaluated with stereoscopic photo or biomicroscopy using a contact lens to detect retinal thickening with or without hard exudates [4,76]. Nowadays, OCT is considered as an essential adjuvant for the early detection of retinal thickening [4]. Furthermore, fluorescein angiography (FA) is widely utilized to differentiate between vascular anomalies, such as intraretinal microvascular abnormalities (IRMA) and neovascularization (NV), an important step for the diagnosis of PDR. Therefore, OCT and FA have been used interchangeably with fundus examination in clinical practice.

Imaging techniques are rapidly evolving across most medical fields, and their results are usually assessed by experienced physicians in a process called observer-driven pattern recognition [77]. However, the evidence-based practice often requires standardized and unbiased procedures in order for conclusions to be drawn objectively [77]. Thus, many score systems have been developed to guide imaging interpretation in a semi-automatic manner [77]. Although the term biomarker can refer to laboratory-related characteristics, the wider definition includes anatomical and imaging findings [14]. Imaging biomarkers are often quantitative measurements and findings, which can enhance our ability for decision making in an automated manner [77]. Furthermore, progression in the field of machine learning and artificial intelligence allows us to exploit imaging biomarkers more than ever [77]. Consequently, many imaging biomarkers can be identified among literature in the field of retinal disorders [78].

### 5.1. Optical Coherence Tomography Biomarkers

OCT is an imaging modality that utilizes low coherence interferometry principles to produce an in vivo cross-sectional depiction of the neurosensory retina and choroid, by processing the backscattered light [79]. Macular edema can occur in many retinal diseases, and clinicians often face difficulties in differentiating between concomitant pathologies, like pseudophakic cystoid macular edema (PCME) and DMO [80]. OCT biomarkers can provide objective measurements in such cases, aiding objective diagnosis and therapeutic management, accordingly [80]. Furthermore, subtle changes can be detected long before they are evident in slit lamp biomicroscopy [81]. As multiple mechanisms are involved in DR, it has been proposed that many different phenotypes of DMO could be identified using multimodal imaging, pointing to a future of personalized management [82]. The proposed OCT biomarkers in bibliography are presented below, and their various clinical applications are summarized in Table 2.

#### 5.1.1. Macular Thickness and Volume

The pachymetry maps of the retina are integrated in almost every modern OCT device [83]. Thickness maps have been used in everyday practice to monitor responses to therapy such as anti-VEGF agents; however, recent data suggest that the anatomical alterations are not always translated in the functional outcomes of visual function [84,85,86]. The distance between vitreoretinal surface and retinal pigment epithelium is detected and defined as retinal thickness; thus, the retinal thickness can be affected by either intra- or sub-retinal fluid accumulation [83]. Additionally, further distinction regarding the distribution of the accumulated fluid involves an initial intracellular component due to cytotoxic degenerative alterations, which gradually lead to extracellular accumulation, caused by the vasogenic disruption of the blood–retina barrier [87]. The complexity and overlaying mechanisms of diffuse retinal thickening point to the need for more specific biomarkers to aim clinical decisions and therapy prognosis [88].

#### 5.1.2. Disorganization of Retinal Inner Layers (DRIL)

**Figure 1:**
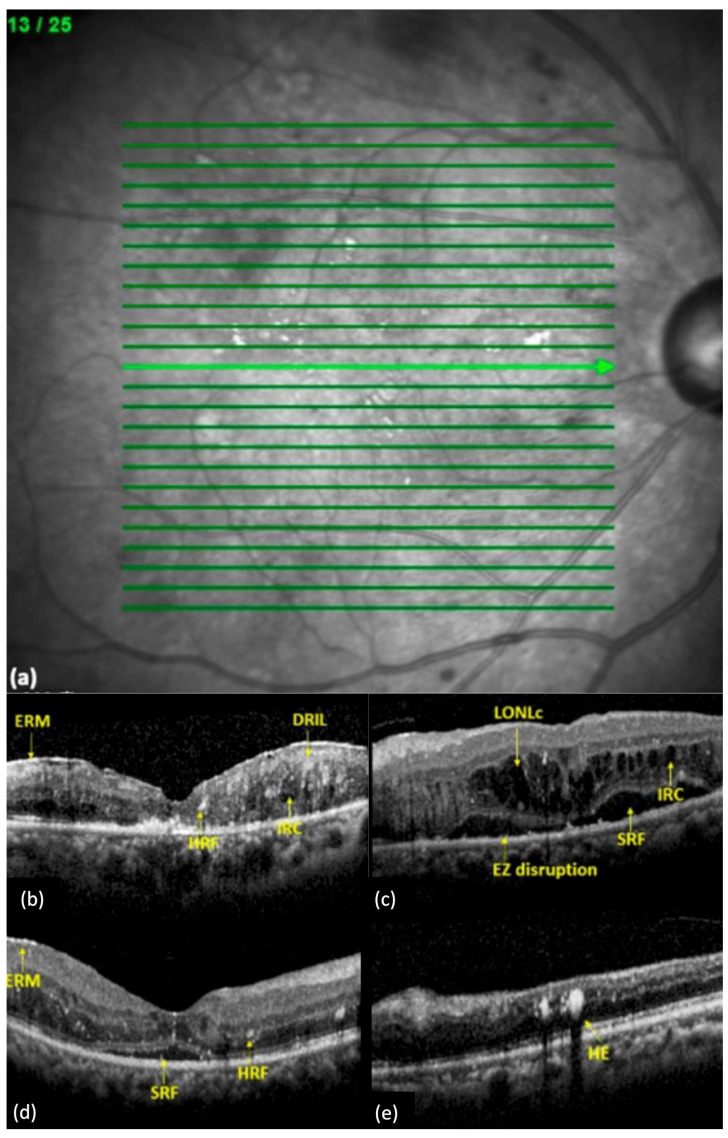
OCT biomarkers in diabetic macular edema (DME) eyes [114]. (**a**) A representative image of a 25–26 mm macular scans centered on the fovea by spectral domain OCT (SD-OCT). (**b**) An OCT of a patient with DME showing the presence of ERM, HRF, intraretinal cyst (IRC), and disorganization of the retinal inner layers (DRIL). (**c**) An OCT of a patient with DME showing the presence of a large outer nuclear layer cyst (LONLC), ellipsoid zone disruptions (EZD), SRF, and IRC. (**d**) An OCT of a patient with DME showing the presence of epiretinal membrane (ERM), subretinal fluid (SRF), and hyperreflective foci (HRF) marked with yellow arrows. (**e**) An OCT of a patient with DME showing the presence of a hard exudate (HE).

DRIL was firstly described as a potential biomarker by Soliman et al. [85], and its current definition is described as the inability to distinguish between the ganglion cell layer–inner plexiform layer complex, inner nuclear layer, and outer plexiform layer (Figure 1b) [89]. It is considered to reflect functional damages in Müller cells, suggested by the presence of related soluble biomarkers, such as GFAP, as discussed previously [89]. DRIL has been proposed as a predictor of visual acuity in eyes with DMO or resolved DMO and has been correlated with disease severity [90,91]. Furthermore, DRIL can be considered as an early sign of retina dysfunction, even in the absence of DMO, in agreement with the speculation of early neurodegeneration involved in DR [92]. In fact, DRIL can have applications on many other vitreomacular, inflammatory, and vascular conditions affecting the macula [93,94,95].

#### 5.1.3. Intraretinal Cystoid Spaces

Cystoid macular edema (CME) is a specific pattern of macular edema caused by various retinal diseases and conditions, such as uveitis, DR, post-surgical inflammation, and age-related macular degeneration [96]. Due to different underlying pathophysiologies, the imaging findings can differ. For instance, the presence of solely retinal cysts is observed in post-cataract macular edema; whereas, in DR, cysts are accompanied by diffuse thickening (Figure 1b). Furthermore, cysts are initially present in the deeper outer nuclear layers, where microaneurysm related leakage is initiated [80]. Additionally, DR-related CME usually spares the central fovea, and foveal depression is usually maintained [80]. When the size of the cyst exceeds 220 μm, the negative impact on the visual outcome is expected to be more extensive, as cysts in the deeper layers observed in DMO are believed to cause direct damage to the photoreceptors (Figure 1c) [97]. An interesting finding regarding the reflectivity of the content of these cysts was described by Liang et al. They assume that hyper-reflective and solid-appearing cysts are remnants of fluid-filled cysts, related to a poor prognosis and lack of response to treatment [98].

#### 5.1.4. Bridging Retinal Processes

When the accumulation of cysts and edema exceed the stretching durability of the retina, permanent damage may be established to sensitive bipolar neuron axons [88]. Residual vertical retinal tissue spanning the retinal thickness has been proposed as an indicator of remaining function despite the presence of large cysts and advanced thickening [99]. The presence of these bridging retinal processes can predict an adequate response to the anti-VEGF agents in terms of visual acuity after the resolution of edema [99]. To avoid misconceptions, it is essential that clear definitions be adopted in the future.

#### 5.1.5. Subfoveal Neurosensory Detachment

The absence of subretinal fluid is considered a distinctive characteristic of macular edema due to diabetic retinopathy [80]. However, ME with SND is one of the phenotypes of DME, accounting for 15–30% of the cases (Figure 1c,d) [100]. The studies about the role of SND in DME are controversial. Vujocevic et al. described SND as an indicator of retinal impairment associated with poorer visual outcomes, while Gerendas et al. reported a better response to anti-VEGF therapy and improved final outcomes [100,101]. A better interpretation of the study by Gerendas, in a post hoc analysis, would explain that, even if the presence of SND is a negative predictive factor when left untreated, the response to anti-VEGF therapy should be considered greater [102]. Consequently, the resorption of subretinal fluid could be considered a therapeutic target as well [102].

#### 5.1.6. Hyperreflective Retinal Foci (HRF) and Hard Exudates

HRF appear as intraretinal hyperreflective spots and can refer to multiple entities of different origin [103]. Vujosevic et al. attempted to categorize these findings according to their size, location, and imaging properties [103]. An HRF < 30 μm, reflectivity similar to nerve fiber layer, and the absence of back shadowing may supposedly consist of aggregates of microglial cells, not clinically apparent in biomicroscopy (Figure 1d), while an HRF > 30 μm, reflectivity similar to retinal pigment epithelium–Bruch complex, presence of back shadowing, and location in the outer retina may represent hard exudates (Figure 1e).

On the contrary, an HRF > 30 mm, the presence of back shadowing, and location in the inner retina may represent microaneurysms. Both microaneurysms and hard exudates correspond to clinically evident findings in biomicroscopy and en face OCT [103]. Hard exudates have been a hallmark clinical sign of diabetic retinopathy and are associated with high serum lipid levels and worse vision prognosis [104]. They are believed to consist of lipo-proteinaceous deposits leaking from damaged capillaries [105]. A study by Shin et al. suggested that the presence of hard exudates is a prognostic factor for a more favorable response to intravitreal steroids than anti-VEGF agents [106].

A study by Lee et al. demonstrated elevated inflammation markers in eyes with HF, indicating their use as an inflammation biomarker [107]. Hence, they have been speculated to be a possible biomarker for predicting improved response in intraocular steroid implants [108]. Nevertheless, another study by Kim et al. demonstrated higher recurrence rates in eyes with higher numbers of HRF on OCT [109]. It is worth mentioning that HR size was defined as being between 20 and 40 μm. It is of great importance that the overlapping role of these imaging findings will be distinguished in order to define specific guidelines with possible application in personalized medicine based on imaging biomarkers.

#### 5.1.7. Choroidal Biomarkers

Advances in OCT technology, with the introduction of enhanced depth imaging optical coherence tomography (EDI-OCT), have enhanced the evaluation of choroidal diseases in the pachychoroid spectrum, as well as neoplasms of the choroid [110]. Subfoveal choroidal thickness was shown to predict the visual outcome and response to anti-VEGF therapy in naïve eyes with DME. Agrawal et al. described this as the ratio of choroidal luminal area to total choroidal area, suggesting it as a novel biomarker for the functional evaluation of choroid [111]. It is believed to be a more objective biomarker for monitoring disease progression, as it is correlated with the severity of DR [112]. Last but not least, choroidal hyperreflective foci, described as spots of higher reflectivity within the choroid and, likewise, HF in the retina, are presented as novel prognostic factors of poor visual outcome [113].

### 5.2. Fluorescein Angiography Biomarkers

Despite its invasive nature and time-consuming procedure, fluorescein angiography (FA) is a key diagnostic tool in the diagnosis and staging of diabetic retinopathy. In recent years, numerous biomarkers for DR have been identified through fluorescein angiography. For instance, the duration of DR has been shown to correlate with disease progression, with each additional year of diagnosis associated with a 10.75 mm^2^ increase in total non-perfusion (NP) area. Sex and skin color has been reported to exhibit influence, with females having a greater area of non-perfusion in the posterior pole than males, while black patients exhibit a significant extensive NP compared to white patients [115].

Furthermore, microaneurysms may not be visible in the fundoscopy and can be detected as punctate areas of hyperfluorescence in the FA. Macular ischemia can be indicated through an extension of FAZ, which is extensive in eyes due to perifoveal capillary occlusion [75]. Fluorescein leakage is also a crucial biomarker, particularly in cases of neovascularization, where leakage can obscure the visualization of new blood vessels. Microaneurysms cause focal leakage, leading to localized macular edema, while more diffuse leakage resulting from capillary bed disruption can cause widespread macular edema [116]. FA is also essential for detecting and differentiating intraretinal microvascular abnormalities (IRMAs) [117].

### 5.3. OCT Angiography Biomarkers

OCT angiography (OCTA) has many advantages when compared with traditional forms of imaging. It provides similar information as FA about retinal vasculature, such as areas of non-perfusion and neovascularization, without the need for contrast dye; hence, it is considered a non-invasive procedure [118]. The main advantage of OCTA is its ability to distinctly analyze each of the retinal capillary plexuses, which is vital for understanding the pathophysiologic changes in DR compared to the en face depiction of FA [118]. Although OCTA produces static images only, important information about the blood flow in the retinal circulation can be extracted [118].

The drawbacks of OCTA include the narrow field of view and its susceptibility to artifacts, which are gradually improved with the continuous advancement of the technology, like the introduction of ultra-wide-field OCTA [118]. The future clinical use of wide-field OCTA could be used to monitor peripheral NV and its response to treatments, like panretinal photocoagulation, with a view to gradually replacing FA, which still remains the gold standard in this aspect [119]. Various OCTA-related quantitative biomarkers have been proposed for future clinical applications. These biomarkers, as well as their clinical applications, are summarized in Table 2. Deep capillary plexus (DCP) has been shown to be the first capillary plexus to be affected in DR using OCTA technology [120].

#### 5.3.1. Retinal Vascular Density (VD)

Vessel density (VD) is the ratio of blood vessel area to the total area under measurement, displaying high reproducibility rates [121]. A study by AttaAllah et al. described that VD in the deep capillary plexus (DCP) is significantly reduced in eyes with DR, which may be utilized in the risk assessment of the visual outcome, as well as the monitoring of the response to treatment [122]. Improvement in VD after dexamethasone implant therapy is also reported by Toto et al. [123].

Notably, at the early preclinical stage, capillary density can even increase as a compensation response to the increased metabolic demands of the affected retina [124]. However, a study by Carnevali et al. reported reduced density in the DCP as a very early sign of DR, before clinically apparent changes occur [125]. Interestingly, only type 1 DM patients were included in this study, which can be considered an ideal population for studying DR biomarkers, as the confounding effect of other systemic factors other than diabetes is avoided.

#### 5.3.2. Foveal Avascular Zone (FAZ)

The same study by AttaAllah also concluded the already known fact that FAZ was significantly larger at the level of the superficial capillary plexus (SCP) in eyes with DR, which correlated with worse prognosis [122].

Furthermore, Takase et al. highlighted the importance of enlarged FAZ as a potential biomarker for the early detection of DR [126]. As mentioned earlier, in the early stage of DR, neurodegenerative changes have been shown to precede vascular changes.

However, FAZ enlargement may occur as early as at the thinning of the initial inner layers, as has been proposed by Kim et al. [127,128]. Additionally, Kim et al. have further reported an association between FAZ enlargement and neurodegeneration, suggesting these as potential biomarkers for disease progression [129].

However, FAZ is a variable parameter, even among healthy individuals, meaning that the careful application of OCTA results is required. FAZ may be a clinically relevant biomarker in advanced disease [120].

#### 5.3.3. Fractal Dimension (FD)

Fractal dimension analysis consists of complex estimations of the microvascular and geometric alterations of the retinal vessels. FD is significantly reduced in eyes with DR; however, the correlation with disease severity is vague [130]. Zahid et al.’s study supports that there is not a connection between disease severity, while Tang et al. report that FD was affected with disease progression [120,130]. However, FD can be affected by many other risk factors, such as aging and obesity, so careful interpretation is required before possible clinical use [120].

#### 5.3.4. Intercapillary Spacing

The intercapillary spacing is a novel biomarker aiming to evaluate subtle changes in microcirculation, which may arise at the very early stages of the disease [131]. The area is similar to capillary non-perfusion, which refers to larger areas of decreased blood flow. Terada et al. supported in their published results that it can be utilized for the early detection of DR, as well as for the disease severity assessment [131].

## 6. The Role of Artificial Intelligence

Over the last decade, there has been a remarkable surge in the application of artificial intelligence in ophthalmology. A plethora of convolutional neural networks (CNNs) has been created for the automated diagnosis of various retinal diseases, spanning both medical and surgical conditions [132]. The integration of neural networks into daily clinical practice has the potential to significantly reduce examination times, while improving access to public health resources. Furthermore, advancements in artificial intelligence could lead to the further expansion of telemedicine, enabling patients in rural areas to access specialized care. This would facilitate the timely and accurate diagnosis of diabetic retinopathy, ultimately reducing the burden of preventable vision loss.

Sandhu et al. developed and trained a neural network for the automated diagnosis of diabetic retinopathy [133]. For OCT imaging, the selected clinical biomarkers were reflectivity, curvature, and retinal thickness. For OCT-A, the following biomarkers were selected: blood vessel caliber, vessel density, the size of the foveal avascular zone (FAZ), and the number of bifurcation and crossover points. The system achieved an overall accuracy of 96%, with a sensitivity of 100%, a specificity of 94%, and an AUC of 0.96. Gargeya and Leng used 75.137 fundus images to train an algorithm. Lesions, such as retinal hemorrhages, neovascularizations, and hard exudates, were implemented as biomarkers for the detection of diabetic retinopathy. This algorithm achieved an area under the receiver operating characteristic curve (AUC) of 0.97, with a sensitivity of 94% and a specificity of 98%.

In addition, artificial intelligence is anticipated to play a transformative role in advancing personalized medicine by incorporating each patient’s unique characteristics. These factors include the type and duration of diabetes, trends in hemoglobin A1c levels, kidney function, current retinal findings, and retinal vascular morphology [134]. By leveraging this comprehensive, AI-driven approach, the management of DR has the potential to be revolutionized, paving the way for truly personalized treatment.

## 7. Electroretinography (ERG)

Most imaging techniques evaluate structural changes in either the anatomy of the retinal layers or the microvasculature. On the contrary, electroretinogram can reflect functional changes occurring in the early stages [135].

Signal attenuation in light-adapted single-flash and flicker ERGs can be detected in diabetic patients without clinical DR, showcasing early cone dysfunction [136]. Pattern ERG, on the other hand, can highlight dysfunction of the inner layers in eyes with mild NPDR, as showed by Park et al., further supporting the neurodegeneration hypothesis of DR [137].

Multifocal ERG has been shown to detect abnormal patterns as a delay in N1 and P1 components in patients without the clinical signs of DR [138]. Moreover, multifocal ERG could be used to assess the macular function after panretinal photocoagulation and serve as a predictor of poor visual outcomes [139].

However, some major limitations of ERG application in clinical practice include the lack of standardized protocols among different studies, as well as the time-consuming nature of the examination, hindering its wide integration into the clinical practice [140]. Interestingly, hand-held devices can be less consuming and equally effective in the future for the improvement of functional DR change assessment in the clinical routine [141].

## 8. Conclusions

The era of precision medicine and evidence-based clinical practice requires strict guidelines and objective tools to enhance decision making for the benefit of patients. Biomarkers are characteristics aiming to improve our ability to quantify and standardize the process of interpretation of laboratory and imaging findings. The management of diabetic retinopathy can be greatly improved with the recent advances in imaging and the analytical techniques of the last decades. Furthermore, the development of the multi-omics field promises to facilitate the discovery of many novel laboratory-related biomarkers in blood and ocular matrices. Meanwhile, artificial intelligence is growing exponentially, and big data analysis from future clinical studies can lead to the automated and objective clinical application of modern imaging techniques.

The main purpose of this review was to present the vast field of systemic and ocular biomarkers, regarding diabetic retinopathy and diabetic macular edema. We tried to include all the novel biomarkers under evaluation in an up-to-date manner. However, the limitations of these studies include the huge amount of data among the literature for both soluble and imaging biomarkers, which should be summarized for the reader.

The study of molecular biomarkers has not reached clinical application, with the exception of HbA1c, which has been the gold standard for the risk assessment of DR. Multi-omics has elucidated our understanding for the pathogenesis of DR, through the discovery of many molecules involved in the disease mechanisms. However, these molecules lack specificity, due to their limited contribution in the numerous and complex pathways of a multifactorial disease, such as DR. Moreover, the detection of soluble biomarkers by multi-omics techniques is further complicated by the presence of high-abundance molecules, like albumin and globulins, especially in human plasma. Ideally, in the near future, novel soluble biomarkers will facilitate the detection of the prevalent underlying pathogenesis for each patient, promoting personalized medical management.

Although vitreous humor is ideal for biomarker studies, it requires invasive collection methods, limiting its widespread applicability. Thus, a shift in our interest towards ocular tissues requiring minimally invasive techniques of collection, such as tears, has emerged. However, there is not a clinically established method for tear collection, which should be addressed before possible tear biomarkers are integrated in routine clinical practice. More especially, significant variability exists among biomarker studies currently, making it challenging to create universally accepted clinical applications.

On the other hand, physicians are more familiar with most of the imaging biomarkers referred to in this review. In fact, many of them have already been used in clinical practice as medical signs traditionally. However, most of these imaging findings have recently acquired the quantitative role of biomarkers in order to facilitate early prognosis, disease progression, and therapy monitoring, as well as the personalized selection of the already established therapeutic options for DR. Nevertheless, future clinical trials should be conducted to fully establish the role of these biomarkers in evidence-based decision making.

## Figures and Tables

**Table 1:** Categories of molecular/soluble biomarkers classified based on their site.

	Vitreous	Aqueous	Tears	Serum
Inflammatory	IL-1 [32] IL-2 [40] IL-4 [40] IL-6 [22,32,41] IL-8 [22,32,33,40,41] IL-13 [40] TNF-α [22,40,41] IFN-γ [40] MCP-1 [22,41] Endothelin ET A [41] Endothelin ET B [41] Platelet-derived growth factor BB chain PDGF-BB [41] Pigment epithelium-derived factor PEDF [41] Erythropoeitin EPO [40,41] Eotaxin [40] Hepatocyte growth factor HGF [41] Inositol 1,4,5-trisphosphate receptor type 2 (ITPR2) [38] Calcium homeostasis endoplasmic reticulum protein (CHERP) [38] Coronin-1A (CORO1A) [38] G-CSF [40] GM-CSF [40] ICAM [40] VCAM [40] Complement C1,4,7,8 [32]	IL-1β [34] IL-2 [34] IL-4 [34] IL-5 [34] IL-6 [22,40] IL-8 [22,40] IFN-γ [34] TNF-α [34]	IL-2 [37] IL-5 [37] IL-18 [37] TNF [37] MMP(matrix metalloproteinases)-2,3,9 [37]	CRP [36]
Angiogenesis	VEGF [22,33,40,43,44] Angiopoeitin-2 [40,43] PIGF [22,33,44] ANGPTL-4 [33] Syndecan-1 [33] Placenta growth factor (PlGF) [43] Matrix metalloproteinase-2 (MMP-2) [43] Matrix metalloproteinase-9 (MMP-9) [43] Angiopoietin-1 (Ang-1) [43] Erythropoietin (EPO) [43,46] Transforming growth factor-b1 (TGFb1) [43] Thrombospondin-1 (TSP1) [45]	VEGF [22,34,46] PIGF [22] Erythropoietin (EPO) [46]		
Metabolic	L-Glutamine [48] Citrulline [48] L-Lactic acid [48] L-Glutamic acid [48] Pyruvic acid [48] Acetic acid [48] D-Glucose [48] L-Alanine [48] L-Threonine [48] L-Lysine [48] Galactitol [49] Ascorbic acid [49] Xanthine [49] Proline [49] Pyruvat [49] Pyroglutamic acid [49] Docosatetraenoic acid (DTA) [49] Eicosapentaenoic acid (EPA) [49] Docosahexaenoic acid (DHA) [49] Arachidonic acid (ARA) [49] ±9(10)-dihydroxy-Octadecenoic acid (±9(10)-DiHOME) [49] ±19.20-epoxy-docosapentaenoic acid (±19,20-EpDPE) [49] ±12(13)- epoxy-octadecenoic acid (±12(13)-EpOME) [49]	L-Glutamine [48] Citrulline [48] L-Lactic acid [48] Pyruvic acid [48] D-Glucose [48] L-Lysine [48] L-Alanine [48] L-Threonine [48] Cytidine [49] Adenosine [49] 1,5-gluconolactone [49] 2-deoxyribonic acid [49] Gluconic acid [49] urea [49] Glutamic acid [49] Fumaric acid [49] Uridine [49] Acetic acid [49] Pseudouridine [49] N-acetyltryptophan [49] Leucylleucine [49] Glutamate [49] 3,4-dihydroxybutyric acid (3,4-DHBA) [49]	Acylcarnitines [51] Methionine-sulfoxide [51] Cholesteryl ester 15:1 [51] Eicosadienoic acid [51] Triacylglyceride (14:0_34:0) [51] Triacylglyceride (16:0_32:0) [51] D-Glutamine [52] D-glutamate [52] Azelaic acid + guanosine [52]	L-Glutamine [48] Citrulline [48] L-Glutamic acid [48] Acetic acid [48] AGEs [50] N-Epsilon-carboxymethyl lysine (N-*ε*-CML) [50] Pentosidine [50] 3-deoxyglucosone (3-DG) [50] AGE receptors (RAGE) [50]
Neurodegenerative	neurotrophins [53]	Glial fibrillary acidic protein (GFAP) [54] Aquaporin 1 (AQP1) [54] Aquaporin 4 (AQP4) [54]		
Oxidative stress	Malondialdehyde (MAD) [55] Total antioxidant capacity (TAC) [56] Direct lipid hydroperoxidation (LPO) [55] Glutathione (GSH) Nitric oxide (NO) [41]	Total antioxidant capacity (TAC) [56]	Calmodulin-like protein 5 (CALML5) [37] Glutamine synthetase (GLUL) [37] Protein SET; protein SETSIP (SET/SETSIP) [37] DNA dC->dU-editing enzyme APOBEC-3A (APOBEC3A) [37] Cathepsin L1 (CTSL) [37] Glutaredoxin-1 (GLRX) Nicotinamide phosphoribosyltransferase (NAMPT) [37] Alpha/beta hydrolase domain-containing protein 14B (ABHD14B) [37] Protein disulfide-isomerase A3 (PDIA3) [37] Calmodulin-like protein 3 (CALML3) [37]	Malondialdehyde (MAD) [55] Total superoxide dismutase (SOD) [55]
Non-coding RNA gene transcription factors	Lnc-RNAs MEG3/MALAT1 [57]	miR-200b-3p [58] let-7c-5p [58] miR-365-3p [58] miR-199a-3p [58] Lnc-RNAs MEG3/MALAT1 [57]	miR-218-5p [59]	OSER1 [60] HIPK2 [60] DDRGK1 [60] Lnc-RNAs MEG3/MALAT1 [57]

**Table 2:** OCT biomarkers.

Optical Coherence Tomography (OCT) Biomarkers	Early Diagnosis	Prognosis	Disease Progression	Therapy Response	Personalized Medicine Treatment Selection
Macular thickness and volume [83,84,85,86,87,88]				x	
Disorganization of retinal inner layers (DRIL) [85,89,90,91,92,93,94,95]	x	x	x		
Intraretinal cystoid spaces [80,96,97,98]		x		x	
Bridging retinal processes [88,99]		x			x (anti-VEGF)
Subfoveal neurosensory detachment [100,101,102]		x		x	x (anti-VEGF)
Hyperreflective retinal foci [103,104,105,106,107,108,109]		x			x (steroids)
Subfoveal choroidal thickness [110]		x			x (anti-VEGF)
Choroidal vascularity index [111,112]		x			
Choroidal hyperreflective foci [113]		x			

## Data Availability

The raw data supporting the conclusions of this article will be made available by the authors on request.
